# Association of Adverse Neighborhood Exposures With HIV Viral Load in Pregnant Women at Delivery

**DOI:** 10.1001/jamanetworkopen.2020.24577

**Published:** 2020-11-06

**Authors:** Florence M. Momplaisir, Tanner Nassau, Kari Moore, Clara Grayhack, Wanjiku F. M. Njoroge, Ana V. Diez Roux, Kathleen A. Brady

**Affiliations:** 1 Division of Infectious Diseases, University of Pennsylvania Perelman School of Medicine, Philadelphia; 2 The Leonard Davis Institute of Health Economics, University of Pennsylvania, Philadelphia; 3 AIDS Activities Coordinating Office, Philadelphia Department of Public Health, Philadelphia, Pennsylvania; 4 Urban Health Collaborative, Dornsife School of Public Health at Drexel University, Philadelphia, Pennsylvania; 5 Drexel College of Medicine, Philadelphia, Pennsylvania; 6 Department of Child and Adolescent Psychiatry and Behavioral Sciences, The Children’s Hospital of Philadelphia, Pennsylvania; 7 Department of Psychiatry, University of Pennsylvania Perelman School of Medicine, Philadelphia; 8 Dean’s Office, Dornsife School of Public Health at Drexel University, Philadelphia, Pennsylvania

## Abstract

**Importance:**

Racial disparities in maternal morbidity and mortality are in large part driven by poor control of chronic diseases. The association between adverse neighborhood exposures and HIV virologic control has not been well described for women with HIV during pregnancy.

**Objective:**

To evaluate the association between adverse neighborhood exposures and HIV viral load at delivery.

**Design, Setting, and Participants:**

This population-based cohort study assessed HIV surveillance data for pregnant women with HIV who had live deliveries in Philadelphia from January 1, 2005, through December 31, 2015. Data analyses were completed in August 2020.

**Exposures:**

Neighborhood exposures included extreme poverty, educational attainment, crime rates (using separate and composite measures), and social capital categorized above or below the median. Each neighborhood exposure was modeled separately to estimate its association with elevated HIV viral load.

**Main Outcomes and Measures:**

The main outcome was elevated HIV viral load of ≥200 copies/mL at delivery. We hypothesized that adverse neighborhood exposures would be associated with higher odds of having an elevated viral load at delivery. Confounders included birth year, age, race/ethnicity, previous birth while living with HIV, and prenatal HIV diagnosis. Prenatal care and substance use were considered potential mediators. We used logistic mixed effects models to estimate the association between neighborhood exposures and elevated viral load, adjusting for confounders in Model 1 and confounders and mediators in Model 2.

**Results:**

There were 905 births among 684 women with HIV, most of whom were aged 25 to 34 years (n = 463 [51.2%]) and were Black non-Hispanic (n = 743 [82.1%]). The proportion of women with elevated viral load decreased from 58.2% between 2005 and 2009 to 23.1% between 2010 and 2015. After adjusting for confounders in Model 1, higher neighborhood education was associated with lower odds of having an elevated viral load (adjusted odds ratio [AOR], 0.70; 95% CI, 0.50-0.96). More violent crime (AOR, 1.51; 95% CI, 1.10-2.07), prostitution crime (AOR, 1.46; 95% CI, 1.06-2.00), and a composite measure of crime (AOR, 1.44; 95% CI, 1.05-1.98) were positively associated with having a higher HIV viral load. These associations remained after adjusting for mediators in Model 2. In addition, the AOR for intermediate prenatal care varied between 1.93 (95% CI, 1.28-2.91) and 1.97 (95% CI, 1.31-2.96), whereas the AOR for inadequate prenatal care varied between 3.01 (95% CI, 2.05-4.43) and 3.06 (95% CI, 2.08-4.49) across regression models.

**Conclusions and Relevance:**

In this cohort study, adverse neighborhood exposures during pregnancy and poor engagement in prenatal care were associated with poor virologic control at delivery. These findings suggest that interventions targeted at improving maternal health need to take the social environment into consideration.

## Key Points

**Question:**

Are adverse neighborhood exposures associated with poor virologic control of HIV in pregnant women at labor and delivery?

**Findings:**

In this cohort study of 905 births among 684 women with HIV, women residing in neighborhoods with high rates of violent and prostitution crimes were more likely to have poor virologic control, whereas women residing in neighborhoods with high rates of education were more likely to have better virologic control.

**Meaning:**

These findings suggest that social determinants need to be addressed to improve maternal health.

## Teaser

This cohort study evaluates the association between adverse neighborhood exposures and virologic control in pregnant women with HIV at labor and delivery.

## Introduction

Maternal mortality is a serious threat to public health in the United States. In 2016, the pregnancy-related mortality rate was 16.9 deaths per 100 000 live births, an increase from 7.2 deaths per 100 000 live births in 1987.^1^ Racial/ethnic disparities exist, with Black women being 3 times more likely to be affected compared with White women.^2^ Maternal death can occur because of complications directly related to birth; however, the majority of deaths are associated with poor control of chronic diseases.^3^ Pregnant women with HIV tend to experience multimorbidity: in a population-based cohort of 7 772 999 births (2003-2011), births among women with HIV (n = 1997) were associated with a higher prevalence of diabetes, hypertension, and prenatal drug and alcohol use.^4^ Women with HIV had significantly higher birth complications, including venous thromboembolism, postpartum hemorrhage, and postpartum sepsis, and they experienced higher maternal mortality compared with women without HIV (odds ratio, 21.52, 95% CI, 12.96-35.72).^4^ A systematic review and meta-analysis of studies in the early antiretroviral therapy (ART) era found that the pooled risk ratio for maternal mortality for women with HIV (vs without) was 7.74 (95% CI, 5.37-11.16).^5^ A recent study done in the current ART era estimates this risk to be 5 times higher for women with HIV compared with women without HIV.^6^

Factors at different levels are likely driving the association between HIV and maternal mortality. We know that HIV itself, even in the setting of viral suppression, creates a chronic inflammatory state, putting women at increased risk for adverse pregnancy outcomes.^7,8,9^ However, women with HIV encounter chronic stress in their environment (due to poverty, experiences of racism, and chronic exposure to adverse social determinants),^10^ and the impact of this stress on maternal health is less understood. We addressed this by studying the association between adverse neighborhood exposures during pregnancy and having poor virologic control at delivery. We hypothesized that pregnant women with HIV living in neighborhoods with high poverty, high crime, lower education, and lower social capital would be less likely to achieve virologic control at delivery.

## Methods

### Conceptual Framework

In order to understand how neighborhood exposures may affect virologic control at delivery, we used the ecological model of health behavior (Figure). The ecological model of health behavior^11^ is a multilevel model that incorporates contextual determinants of health services use and helps to understand how factors, at multiple levels, can affect health outcomes. Although individual^12,13,14,15^ and interpersonal factors^16,17,18^ associated with virologic control have been well described in the literature, studies on neighborhood exposures are lacking. We hypothesized that adverse living conditions associated with neighborhood poverty, crime, low education, and poor social capital contribute to an environment where chronic stress, through the overactivation of the stress response system, negatively affects the mother, leading to the development of chronic diseases or poor management of existing conditions during pregnancy. There is also an increased risk to the developing fetus through epigenetics.^19^ At the societal level, policies that affect socioeconomic advancement for women and access to equitable sexual and reproductive health likely influence women’s outcomes at birth but are not within the scope of this project.

**Figure.**
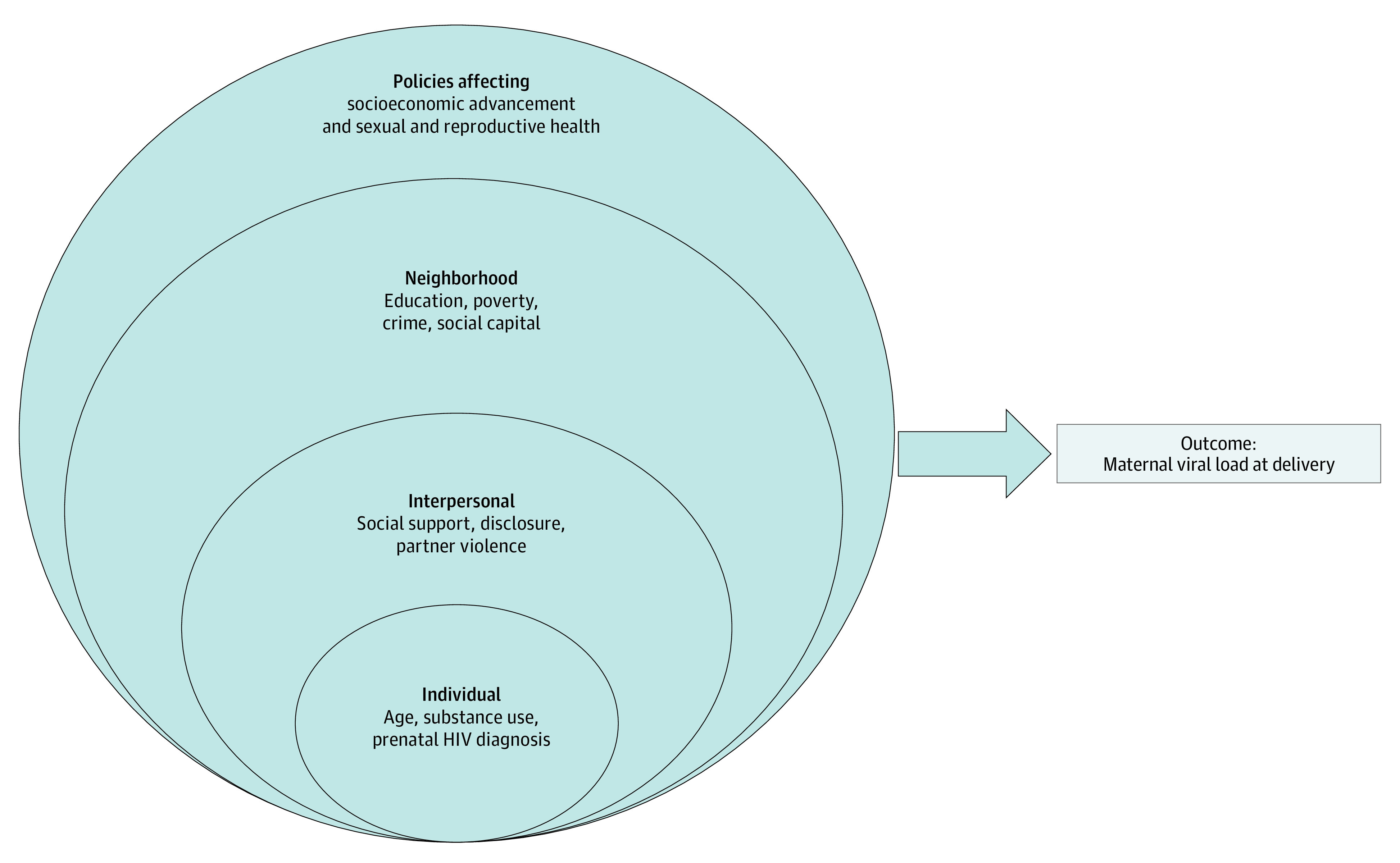
Ecological Model of Health Behavior and Its Application to Maternal Viral Load at Delivery

Study approval and exemption from informed consent (owing to the use of deidentified data) were obtained from the Drexel University and the Philadelphia Department of Public Health institutional review boards. All data for this study were generated using strict adherence to the Centers for Disease Control and Prevention guidelines for security and confidentiality of HIV/AIDS surveillance data. This study followed the Strengthening the Reporting of Observational Studies in Epidemiology (STROBE) reporting guideline.

### Study Population and Data Sources

The study population included pregnant women with HIV with deliveries in Philadelphia over an 11-year period (January 1, 2005, through December 31, 2015). Demographic and clinical data during pregnancy were abstracted from the Perinatal HIV Exposure Reporting (PHER) program. The PHER program is a population-based surveillance system of pregnant women with HIV, conducted through the Philadelphia Department of Public Health as part of a Centers for Disease Control and Prevention surveillance cooperative agreement. Additional clinical variables were obtained by merging PHER with the Enhanced HIV/AIDS Reporting System, a surveillance system of all reported HIV/AIDS cases in Pennsylvania; the combined data set contained demographic and clinical information on pregnant women with HIV. Because PHER and the Enhanced HIV/AIDS Reporting System provide ongoing surveillance, they capture the majority of births from pregnant women with HIV, resulting in a cohort that is representative of the study population.

### Neighborhood Exposures

Home address at delivery was obtained using birth records. Address at delivery was geocoded in SAS, version 9.4 (SAS Institute Inc) using the SAS batch geocoding macro, and census tract was assigned using the 2000 and 2010 US Census Bureau TIGER files for Philadelphia County, Pennsylvania, for births occurring from 2005 through 2009 and 2010 through 2015, respectively. Census tract of residence at delivery was used as a proxy for neighborhood in this analysis. Neighborhood measures, including extreme poverty, educational attainment, crime rates, and social capital, were obtained at the census tract level and linked to women with HIV based on census tract of residence. When years of birth did not align with years of neighborhood exposures, births were matched to the closest available year for the neighborhood exposure in question. Neighborhood measures were dichotomized at the median to create high and low measures of each neighborhood exposure.

#### Socioeconomic Status

Census tract–level extreme poverty and educational attainment were obtained from the American Community Survey^20^ 5-year estimates for 2005 through 2009 and 2010 through 2014. Extreme poverty was defined as the proportion of residents with a ratio of income to poverty being less than 50% of the federally defined poverty level (calculated by dividing a family’s income by the family’s poverty income threshold). Educational attainment was assessed as the percentage of adults 25 years or older with a bachelor’s degree or higher.

#### Crime Rates

Crime data were obtained from the Philadelphia Police Department’s Mapping and Analysis Unit via OpenDataPhilly^21,22^ for the years 2006 through 2015. Violent, drug, and prostitution crimes were measured separately. We also created an index variable for crime, which incorporated the 3 crime measures, to assess the aggregated association of crime with virologic control. Violent crimes were defined as incidents of homicides, rapes, aggravated assaults, robberies, and other assaults. Drug crimes included narcotic and drug law violations. Prostitution included incidents of prostitution and commercialized vice. Yearly crime rates per 10 000 persons for violent, drug, and prostitution crimes were calculated by dividing the total number of incidents by the census-tract population retrieved from the American Community Survey 2005-2009 (years 2006-2009) or American Community Survey 2010-2014 (years 2010-2015). To operationalize the crime index, we first standardized each measure of crime to mean (SD) 0 (1). Next, we calculated overall crime as the sum of these 3 standardized terms for each census tract and dichotomized overall crime at the median.

#### Social Capital

Social capital empirical Bayes estimates^23,24^ were derived from the Southeastern Pennsylvania Household Health Survey.^25^ Social capital varied from 0 to 3, with higher scores indicating higher social capital, and was based on the responses to the following questions and statements: “how likely are people in your neighborhood willing to help others,” “most people in my neighborhood can be trusted,” and “I feel that I belong and am a part of my neighborhood.” Births were matched to the closest preceding social capital.

### Individual-Level Variables

Individual-level variables were classified a priori into confounders and potential mediators based on theory and prior work. Confounders included age, race/ethnicity, and clinical variables (previous birth while living with HIV, prenatal HIV diagnosis, and birth year). Potential mediators included adequacy of prenatal care and prenatal substance use, based on prior work linking these variables to both neighborhood factors and viral suppression.^12,26,27,28,29^ Adequacy of prenatal care was measured by the Kessner Institute of Medicine Index,^30^ a validated index that includes trimester of entrance into prenatal care, number of prenatal visits attended, and gestational age at delivery. Adequate prenatal care was characterized by first-trimester entrance into prenatal care and at least 9 prenatal visits in a full-term pregnancy. Inadequate prenatal care was defined by first prenatal visit in the third trimester or less than 5 visits after 18 weeks of gestation. Any other combination of presentation to prenatal care, number of prenatal visits, and gestational age at delivery was classified as intermediate prenatal care. Prenatal substance use (yes or no) was captured through review of urine toxicology screening, self-report, and social work records and included the use of controlled and uncontrolled substances other than tobacco.

### Outcome Variable

Elevated HIV viral load at delivery was defined as having a viral load of at least 200 copies/mL. When the viral load was lower than 200 copies/mL, women were considered virally suppressed (reference group). The majority of viral loads were obtained during the second or third trimesters with a mean (SD) of 8 (9.2) weeks before delivery; the 50th percentile was 5 weeks.

### Statistical Analysis

Data analyses were conducted in August 2020. We used Pearson χ^2^ tests to compare neighborhood exposures in census tracts where pregnant women with HIV resided at birth to other census tracts in Philadelphia and to compare individual and neighborhood variables for women with elevated vs suppressed HIV viral load. For statistical models (Models 1 and 2), we used logistic mixed effects models with 2 random intercepts—an intercept for census tract to account for clustering in neighborhoods and an intercept for individual to account for mothers with multiple births in the time period. Because of the high correlation between neighborhood measures, we modeled each neighborhood exposure separately. Model 1 adjusted for individual-level confounders. Model 2 added adjustment for potential mediators, namely, prenatal substance use and adequacy of prenatal care. Analyses were performed using SAS, version 9.4 (SAS Institute Inc). *P* values were 2-sided, and *P* < .05 was used to indicate statistical significance.

## Results

There were 944 births among 706 women with HIV between 2005 and 2015. Among all births, 36 had a missing Kessner index, and 3 had missing neighborhood variables. These observations were removed from the sample, resulting in a sample size of 905 births from 684 women with HIV, most of whom were between the ages of 25 and 34 years (n = 463 [51.2%]), Black non-Hispanic (n = 743 [82.1%]), and diagnosed with HIV prior to the index pregnancy (n = 699 [77.2%]). Births with and without missing variables did not differ from the rest of the sample by age, race/ethnicity, and other individual-level characteristics.

We compared characteristics of census tracts with births from women with HIV (n = 187 in 2005-2009 and n = 189 in 2010-2015) with census tracts without births from women with HIV (n = 167 in 2005-2009 and n = 181 in 2010-2015). Here, the unit of analysis was at the census tract, not the individual, level. Census tracts where women with HIV resided had significantly greater extreme poverty (above the median for extreme poverty for 2005-2009, 76 tracts [40.6%] vs 31 tracts [18.6%]; for 2010-2015, 90 tracts [47.6%] vs 36 tracts [19.9%]; *P* < .0001), lower education (below the median for persons with a bachelor’s degree for 2005-2009, 87 tracts [46.5%] vs 29 tracts [17.4%]; for 2010-2015, 82 tracts [43.4%] vs 23 tracts [12.7%]; *P* < .001), and higher crime (eg, above the median for violent crimes per 10 000 population for 2005-2009, 92 tracts [49.2%] vs 38 tracts [22.8%]; for 2010-2015, 76 tracts [40.2%] vs 21 tracts [11.6%]; *P* < .001) and predominantly comprised non-Hispanic Black residents (≥50% non-Hispanic Black for 2005-2009, 112 tracts [59.9%] vs 37 tracts [22.2%]; for 2010-2015, 109 tracts [57.7%] vs 42 tracts [23.2%]; *P* < .001), compared with census tracts without births from women with HIV. These results are included in eTable 1 in the Supplement.

### Unadjusted Associations With HIV Viral Load

Among 905 births, 373 (41.2%) were in women who had an elevated viral load at delivery (Table 1). The proportion of women with elevated viral load decreased from 58.2% between 2005 and 2009 to 23.1% between 2010 and 2015. Women with births occurring between 2010 and 2015 had 79% reduced odds of having an elevated viral load at delivery compared with women with births occurring between 2005 and 2009 (unadjusted odds ratio (UOR), 0.21; 95% CI, 0.16-0.29). Women diagnosed with HIV during (vs before) pregnancy (UOR, 2.11; 95% CI, 1.50-2.97) and women receiving intermediate (UOR, 2.22; 95% CI, 1.53-3.22) or inadequate (UOR, 2.77; 95% CI, 1.98-3.88) vs adequate prenatal care had 2-3 times higher of odds of having an elevated viral load at delivery. Women living in neighborhoods above the median in violent crimes (UOR, 1.56; 95% CI, 1.17-2.09), drug crimes (UOR, 1.59; 95% CI, 1.19-2.13), and prostitution crimes (UOR, 1.60; 95% CI, 1.19-2.14) had higher odds of having an elevated viral load at delivery compared with women in neighborhoods with rates below the median. Education, social capital, and extreme poverty were not associated with HIV viral load in unadjusted analyses.

**Table 1.** Individual and Neighborhood Characteristics of Pregnant Women Living With HIV by Elevated Viral Load at Delivery, Perinatal HIV Exposure Reporting, 2005-2015^a^

Characteristic	Total, No. (%)	Elevated viral load at delivery, No. (%)	UOR (95% CI)	*P* value^b^
Total	905 (100.0)	373 (41.2)	NA	NA
Individual-level variables				
Year of birth				
2005-2009	467 (51.6)	272 (58.2)	1 [Reference]	<.001
2010-2015	438 (48.4)	101 (23.1)	0.21 (0.16-0.29)	
Maternal age at delivery, y				
≥35	185 (20.4)	72 (38.9)	1 [Reference]	.67
25-34	463 (51.2)	191 (41.3)	1.13 (0.77-1.65)	
16-24	257 (28.4)	110 (42.8)	1.21 (0.79-1.85)	
Race/ethnicity				
White, non-Hispanic	52 (5.8)	19 (36.5)	1 [Reference]	.74
Black, non-Hispanic	743 (82.1)	309 (41.6)	1.24 (0.66-2.35)	
Hispanic	88 (9.7)	38 (43.2)	1.33 (0.62-2.87)	
Other (multiple races, Asian, or unknown)	22 (2.4)	7 (31.8)	0.82 (0.26-2.57)	
Substance abuse (other than tobacco)				
No or missing^c^	707 (78.1)	283 (40.0)	1 [Reference]	.24
Yes	198 (21.9)	90 (45.5)	1.23 (0.87-1.73)	
Previous births with HIV diagnosis				
None	571 (63.1)	241 (42.2)	1 [Reference]	.38
≥1	334 (36.9)	132 (39.5)	0.88 (0.65-1.18)	
Prenatal diagnosis of HIV				
HIV diagnosis before pregnancy	699 (77.2)	260 (37.2)	1 [Reference]	<.001
During pregnancy	206 (22.8)	113 (54.9)	2.11 (1.50-2.97)	
Adequacy of prenatal care (Kessner index)				
Adequate	399 (44.1)	115 (28.8)	1 [Reference]	<.001
Intermediate	209 (23.1)	99 (47.4)	2.22 (1.53-3.22)	
Inadequate	297 (32.8)	159 (53.5)	2.77 (1.98-3.88)	
Neighborhood-level exposures				
Extreme poverty				.34
≤14.6% in extreme poverty	454 (50.2)	194 (42.7)	1 [Reference]	
>14.6% or more in extreme poverty	451 (49.8)	179 (39.7)	0.87 (0.65-1.16)	
Education				
≤10% persons ≥ bachelor’s degree	495 (54.7)	217 (43.8)	1 [Reference]	.10
>10% persons ≥ bachelor’s degree	410 (45.3)	156 (38.1)	0.79 (0.59-1.05)	
Crimes				
Violent				
≤371 per 10 000 persons	455 (50.3)	163 (35.8)	1 [Reference]	.003
>371 per 10 000 persons	450 (49.7)	210 (46.7)	1.56 (1.17-2.09)	
Drug				
≤104 per 10 000 persons	456 (50.4)	163 (35.7)	1 [Reference]	.002
>104 per 10 000 persons	449 (49.6)	210 (46.8)	1.59 (1.19-2.13)	
Prostitution				
0 per 10 000 persons	533 (58.9)	196 (36.8)	1 [Reference]	.002
>0 per 10 000 persons	372 (41.1)	177 (47.6)	1.60 (1.19-2.14)	
Social capital				
≤1.90	604 (66.7)	255 (42.2)	1 [Reference]	.43
>1.90	301 (33.3)	118 (39.2)	0.89 (0.65-1.20)	

Abbreviations: NA, not applicable; UOR, unadjusted odds ratio.

^a^ HIV surveillance data were obtained from the Enhanced Perinatal Surveillance and HIV/AIDS Reporting System (2005-2015). Neighborhood-level characteristics were obtained from the following data sources: the American Community Survey, OpenDataPhilly Philadelphia Police Department’s Mapping & Analysis Unit, the Southeastern Pennsylvania Household Health Survey. Neighborhood exposures were dichotomized at the median.

^b^ *P* values for χ^2^ tests.

^c^ Twenty-six individuals (2.9%) were missing data for substance abuse.

### Adjusted Associations

After adjusting for year of birth, maternal age, race/ethnicity, previous birth while living with HIV, and prenatal diagnosis of HIV in Model 1, neighborhood education became negatively associated with having an elevated viral load; the adjusted odds ratio (AOR) for having an elevated viral load was 0.70 (95% CI, 0.50-0.96) for women living in neighborhoods above vs below the median of neighborhood education (Table 2). Violent crimes (AOR, 1.51; 95% CI, 1.10-2.07) and prostitution crimes (AOR, 1.46; 95% CI, 1.06-2.00) remained positively associated with having a higher HIV viral load. Higher drug crimes remained associated with higher odds of having an elevated viral load, but the confidence interval overlapped 1 (AOR, 1.32; 95% CI, 0.96-1.82). The crime index remained significantly associated with our outcome after full adjustment: the AOR was 1.44 (95% CI, 1.05-1.98) for women in neighborhoods with higher aggregated crimes compared to women in neighborhoods with lower aggregated crimes. In Model 2, which adjusted for prenatal substance use and adequacy of prenatal care, these associations remained statistically significant.

**Table 2.** Adjusted Odds Ratios of HIV Viral Nonsuppression at Delivery Associated With Neighborhood Exposures, Perinatal HIV Exposure Reporting, 2005-2015^a^

Variable	Model 1, AOR (95% CI)	*P* value	Model 2, AOR (95% CI)	*P* value
Extreme poverty				
≤14.6% in extreme poverty	1 [Reference]	.70	1 [Reference]	.48
>14.6% in extreme poverty	0.94 (0.68-1.29)		0.89 (0.64-1.23)	
Educational level				
≤10% persons ≥ bachelor’s degree	1 [Reference]	.03	1 [Reference]	.03
>10% persons ≥ bachelor’s degree	0.70 (0.50-0.96)		0.69 (0.50-0.96)	
Violent crime				
≤371 crimes per 10 000 persons	1 [Reference]	.01	1 [Reference]	.01
>371 crimes per 10 000 persons	1.51 (1.10-2.07)		1.50 (1.09-2.07)	
Drug crime				
≤104 crimes per 10 000 persons	1 [Reference]	.08	1 [Reference]	.11
>104 crimes per 10 000 persons	1.32 (0.96-1.82)		1.30 (0.94-1.80)	
Prostitution crime				
0 crimes per 10 000 persons	1 [Reference]	.02	1 [Reference]	.03
>0 crimes per 10 000 persons	1.46 (1.06-2.00)		1.44 (1.04-1.99)	
Crime index (violent, drug, prostitution)				
≤Median	1 [Reference]	.02	1 [Reference]	.05
>Median	1.44 (1.05-1.98)		1.38 (1.00-1.91)	
Social capital				
≤1.90	1 [Reference]	.63	1 [Reference]	.72
>1.90	0.92 (0.66-1.29)		0.94 (0.67-1.32)	

Abbreviation: AOR, adjusted odds ratio.

^a^ In Models 1 and 2, each neighborhood exposure is included separately and adjusted for confounders (ie, year of birth, maternal age, race, previous birth while living with HIV, prenatal diagnosis of HIV). Model 2 built on Model 1 and also includes adjustment for potential mediators (ie, prenatal substance use and adequacy of prenatal care). All models adjusted for clustering at the census tract level and for clustering for mothers with multiple births.

### Mediators and Confounders

The association between confounders, mediators, and virologic control at delivery measured in Model 2 is included in eTable 2 in the Supplement. Adequacy of prenatal care was significantly associated with virologic control across regression models, with a dose-response relationship observed between poorer adequacy of prenatal care and having an elevated viral load; the AOR for intermediate prenatal care varied between 1.93 (95% CI, 1.28-2.91) and 1.97 (95% CI, 1.31-2.96), whereas the AOR for inadequate prenatal care varied between 3.01 (95% CI, 2.05-4.43) and 3.06 (95% CI, 2.08-4.49) across regression models. Substance use was not significantly associated with having an elevated viral load.

## Discussion

In this large observational cohort study, we found that lower neighborhood education and higher violent and prostitution crimes were each associated with an elevated HIV viral load at delivery. These associations were seen even as viral suppression improved over time and after adjusting for confounders and mediators, and taking into account clustering at the census tract and individual level (for women with multiple births). As improving maternal health becomes a public health priority, our study adds to the existing body of literature associating adverse neighborhood exposures with poor maternal outcomes^31,32,33^ and raises the need to address social determinants during care management in pregnancy.

One neighborhood exposure with no association with our study outcome was extreme poverty. This is in line with findings from the Chicago Women’s Interagency HIV Study, which found no association between neighborhood poverty and virologic control in a cohort of women with HIV.^34^ This finding could at least partly be explained by the presence of robust wraparound services provided to people with HIV, including access to ART at low or no cost owing to the Ryan White Comprehensive AIDS Resources Emergency Act.^35^ Under this act, quality metrics related to HIV care are routinely reported to local public health departments. This routine reporting is not the case for other chronic medical conditions driving maternal morbidity and mortality, such as hypertension and diabetes. For HIV-specific outcomes, the combination of free access to care during and after pregnancy and the development of potent and well-tolerated ART likely mitigate the negative effects of poverty on viral suppression.

Pregnant women with HIV residing in neighborhoods with higher education levels were significantly less likely to have an elevated viral load at delivery. One potential mechanism for the beneficial association of neighborhood education with viral suppression might be through community connections that influence health-related services utilization, attitudes, and norms.^36^ For example, women might use social networks within their community to receive recommendations about the use of specific clinics or practitioners. This is important during pregnancy, as both viral suppression^12^ and birth outcomes^37^ have been linked to receiving timely prenatal care. Our analysis demonstrates this importance, as poor prenatal care was associated with virologic failure. However, we did not find an association between social capital and virologic control. In the literature, there is substantial variation in the use of social capital measures, and the association with viral suppression showed mixed findings.^38^ As constructed here, social capital provides a global measure of social cohesion, which is not specific to HIV social support. It is possible that women living in neighborhoods with higher education levels particularly seek support for behaviors focused on improving HIV treatment.

We found an association between violent crime, prostitution crime, and the crime index and elevated viral load. One hypothesis is that neighborhoods with elevated rates of crime might create high-stress environments that negatively impact women’s ability to take their ART daily through a series of mechanisms, such as direct victimization, posttraumatic stress, or depression.^39^ In addition, women might not prioritize self-care in the setting of excessive exposure to stressful environments. The prolonged activation of the hypothalamic-pituitary-adrenal stress response system leads to increasing levels of cortisol affecting not only the mother but also the developing fetus. All of these factors together could reasonably contribute to the associations seen. Studies have shown a strong association between neighborhood violent crimes and higher community viral load,^40^ increased risk for HIV,^41^ and poor overall health.^39,41^

### Limitations

Limitations include the fact that we can solely measure cross-sectional associations and cannot determine causation. Our analyses capture global associations of neighborhood exposures; future studies are needed to better understand the mechanisms driving these associations, particularly the link with chronic stress. In addition, we used census tract–level data, which may not fully capture the true extent of a person’s perception of their neighborhood, and we were unable to incorporate perceived or physiologic measures of chronic stress in our analysis. Finally, there may be additional person-level and census tract–level confounders that we are unable to adjust for owing to a lack of available data. Despite limitations of the geographic areas available and the absence of direct measures of potentially important neighborhood-level processes, the systematic way in which area data were collected for the entire population makes census-based measures a valuable resource.

## Conclusions

In summary, this cohort study found that the overlap between HIV (a chronic disease), adverse neighborhood exposures, and pregnancy continues to occur predominantly among racial/ethnic minorities, which may contribute to the persistence of racial disparities in maternal health. Universal access to HIV care and treatment has helped mitigate the negative association of poverty with viral suppression. This mitigation is not the case for many other chronic diseases where poverty limits access to life-saving treatment during and after pregnancy. Living in high-crime environments likely has a deterrent effect on self-care. Improving maternal health requires a paradigm shift in the way we approach women’s health and calls for addressing the negative effects of social determinants on maternal outcomes.

## Notes

Supplement. **eTable 1.** Racial and socio-economic differences between census tracts with births from pregnant women living with HIV compared to all Philadelphia census tracts, Perinatal HIV Exposure Reporting (2005-2015) **eTable 2.** Individual-level confounders and mediators associated with elevated HIV viral load across regression models
