# Supplementary material for: Association of Adverse Neighborhood Exposures With HIV Viral Load in Pregnant Women at Delivery
Source: JAMA Netw Open. 2020 Nov 6;3(11):e2024577. doi: 10.1001/jamanetworkopen.2020.24577 (PMC7648255; doi:10.1001/jamanetworkopen.2020.24577)
Supplement: Supplement. — eTable 1. Racial and socio-economic differences between census tracts with births from pregnant women living with HIV compared to all Philadelphia census tracts, Perinatal HIV Exposure Reporting (2005-2015) eTable 2. Individual-level confounders and mediators associated with elevated HIV viral load across regression models [file jamanetwopen-e2024577-s001.pdf]

Supplemental Online Content

Momplaisir FM, Nassau T, Moore K, et al. Association of adverse neighborhood exposures with HIV viral load in pregnant women at delivery. *JAMA Netw Open*. 2020;3(11):e2024577. doi:10.1001/jamanetworkopen.2020.24577

eTable 1. Racial and socio-economic differences between census tracts with births from pregnant women living With HIV compared to all Philadelphia census tracts, Perinatal HIV Exposure Reporting (2005-2015)

eTable 2. Individual-level confounders and mediators associated with elevated HIV viral load across regression models

This supplemental material has been provided by the authors to give readers additional information about their work.

© 2020 Momplaisir FM et al. *JAMA Network Open*.

eTable 1. Racial and socio-economic differences between census tracts with births from pregnant women living with HIV compared to all Philadelphia census tracts, Perinatal HIV Exposure Reporting (2005-2015)

	Census tracts with births from WLH*, 2005-2009	CT without births from WLH, 2005-2009	p-value	Census tracts with births from WLH, 2010-2015	CT without births from WLH, 2010-2015	p-value***
**Census tracts**	n=187	n=167		N=189	n=181	
**% non-Hispanic black (NHB)**			**<0.0001**			**<0.0001**
<50%NHB	75 (40.1)	130 (77.8)		80 (42.3)	139 (76.8)	
50% or more NHB	112 (59.9)	37 (22.2)		109 (57.7)	42 (23.2)	
**Socioeconomic Status**						
Extreme Poverty			**<0.0001**			**<0.0001**
≤ 14.6% in extreme poverty	111 (59.4)	136 (81.4)		99 (52.4)	145 (80.1)	

© 2020 Momplaisir FM et al. *JAMA Network Open*.

> 14.6% in extreme poverty	76 (40.6)	31 (18.6)		90 (47.6)	36 (19.9)	
Education			**<0.0001**			**<0.0001**
≤10% persons ≥ Bachelor	87 (46.5)	29 (17.4)		82 (43.4)	23 (12.7)	
>10% persons ≥ Bachelor	100 (53.5)	138 (82.6)		107 (56.6)	158 (87.3)	
**Crimes**						
Violent			**<0.0001**			**<0.0001**
≤371 per 10,000	95 (50.8)	129 (77.3)		113 (59.8)	160 (88.4)	
>371 per 10,000	92 (49.2)	38 (22.8)		76 (40.2)	21 (11.6)	
Drug Violation			**<0.0001**			**<0.0001**
≤104 per 10,000	98 (52.4)	140 (83.8)		115 (60.8)	159 (87.9)	
>104 per 10,000	89 (47.6)	27 (16.2)		74 (39.2)	22 (12.1)	

© 2020 Momplaisir FM et al. *JAMA Network Open*.

Prostitution			**<0.0001**			**0.0004**
0 per 10,000	104 (55.6)	133 (79.6)		129 (68.3)	152 (84.0)	
>0 per 10,000	83 (44.4)	34 (20.4)		60 (31.7)	29 (16.0)	
**Social Capital**			**<0.0001**			0.577
≤1.90	117 (62.6)	53 (31.7)		100 (52.9)	101 (55.8)	
>1.90	70 (37.4)	114 (68.3)		89 (47.1)	80 (44.2)	

*WLH: Women living with HIV

**Percentages based on all tracts, and may not add up to 100% due to rounding

***P-values for chi-squared tests

The unit of analysis is the census tract. Only census tracts with data on all neighborhood variables were included.

© 2020 Momplaisir FM et al. *JAMA Network Open*.

eTable 2. Individual-level Confounders and Mediators associated with elevated HIV viral load across regression models

	Model 2 (extreme poverty)	p-value	Model 2 (education)	p-value	Model 2 (violent crimes)	p-value	Model 2 (prostitution crimes)	p-value	Model 2 (drug crimes)	p-value	Model 2 (crime index)	p-value	Model 2 (social capital)	p-value
**Year**		<0.0001		<0.0001		<0.0001		<0.0001		<0.0001		<0.0001		<0.0001
2005-2009	Ref(---)		Ref(---)		Ref(---)		Ref(---)		Ref(---)		Ref(---)		Ref(---)	
2010-2015	0.20 (0.14-0.28)		0.19 (0.14-0.27)		0.20 (0.14-0.28)		0.20 (0.15-0.28)		0.20 (0.15-0.28)		0.20 (0.15-0.28)		0.20 (0.14-0.27)	
**Delivery Age**		0.77		0.74		0.76		0.76		0.76		0.76		0.80
35+	Ref(---)		Ref(---)		Ref(---)		Ref(---)		Ref(---)		Ref(---)		Ref(---)	
16-24	1.07 (0.67-1.73)		1.04 (0.65-1.68)		1.08 (0.67-1.75)		1.08 (0.67-1.74)		1.06 (0.66-1.72)		1.06 (0.65-1.70)		1.07 (0.66-1.71)	
25-34	1.15 (0.75-1.77)		1.16 (0.76-1.78)		1.17 (0.76-1.79)		1.17 (0.76-1.79)		1.16 (0.76-1.78)		1.16 (0.76-1.77)		1.15 (0.75-1.75)	
**Race/Ethnicity**		0.91		0.87		0.94		0.94		0.94		0.93		0.91
White	Ref(---)		Ref(---)		Ref(---)		Ref(---)		Ref(---)		Ref(---)		Ref(---)	
Black	1.29 (0.64-2.61)		1.32 (0.66-2.67)		1.25 (0.62-2.53)		1.23 (0.61-2.50)		1.25 (0.62-2.53)		1.26 (0.62-2.55)		1.29 (0.64-2.60)	
Hispanic	1.31 (0.56-3.06)		1.23 (0.52-2.86)		1.23 (0.53-2.89)		1.22 (0.52-2.84)		1.22 (0.52-2.85)		1.21 (0.52-2.83)		1.27 (0.55-2.96)	
Other	1.34 (0.37-4.85)		1.41 (0.39-5.12)		1.29 (0.35-4.70)		1.32 (0.36-4.80)		1.31 (0.36-4.76)		1.33 (0.37-4.83)		1.30 (0.36-4.68)	
**Substance Use (mediator)**		0.29		0.29		0.34		0.36		0.31		0.35		0.28
No/missing	Ref(---)		Ref(---)		Ref(---)		Ref(---)		Ref(---)		Ref(---)		Ref(---)	
Yes	1.24 (0.83-1.85)		1.24 (0.83-1.84)		1.21 (0.81-1.81)		1.20 (0.81-1.80)		1.22 (0.82-1.82)		1.21 (0.81-1.80)		1.24 (0.84-1.85)	
**Previous birth with HIV**		0.31		0.36		0.37		0.37		0.35		0.36		0.33
None	Ref(---)		Ref(---)		Ref(---)		Ref(---)		Ref(---)		Ref(---)		Ref(---)	
≥1	1.20 (0.84-1.72)		1.18 (0.83-1.69)		1.18 (0.82-1.68)		1.18 (0.82-1.68)		1.19 (0.83-1.70)		1.18 (0.83-1.69)		1.19 (0.84-1.71)	
**Time of Maternal HIV Diagnosis**		0.01		0.01		0.01		0.01		0.01		0.01		0.01
Before pregnancy	Ref(---)		Ref(---)		Ref(---)		Ref(---)		Ref(---)		Ref(---)		Ref(---)	
During pregnancy	1.68 (1.11-2.53)		1.69 (1.12-2.55)		1.70 (1.13-2.57)		1.69 (1.12-2.56)		1.70 (1.12-2.56)		1.70 (1.13-2.57)		1.68 (1.12-2.54)	
**Kessner Index (mediator)**		<0.0001		<0.0001		<0.0001		<0.0001		<0.0001		<0.0001		<0.0001
Adequate	Ref(---)		Ref(---)		Ref(---)		Ref(---)		Ref(---)		Ref(---)		Ref(---)	
Intermediate	1.97 (1.31-2.96)		1.93 (1.28-2.91)		1.95 (1.30-2.94)		1.93 (1.28-2.91)		1.93 (1.28-2.91)		1.95 (1.29-2.93)		1.94 (1.29-2.93)	
Inadequate/No prenatal care	3.06 (2.08-4.49)		3.05 (2.08-4.48)		3.05 (2.08-4.48)		3.06 (2.08-4.49)		3.04 (2.07-4.47)		3.01 (2.05-4.43)		3.03 (2.07-4.45)	

In models 1 and 2, each neighborhood exposure is included separately and adjusted for confounders (i.e. year of birth, maternal age, race, previous birth while living with HIV, prenatal diagnosis of HIV). Model 2 built on Model 1 and also includes adjustment for potential mediators (i.e. prenatal substance use and adequacy of prenatal care). All models adjusted for clustering at the census tract level and for clustering for mothers with multiple births.

© 2020 Momplaisir FM et al. *JAMA Network Open*.
